# Similarities and Differences in Brain Activation Between Patients With Schizophrenia and Obsessive-Compulsive Disorder: A Near-Infrared Spectroscopy Study

**DOI:** 10.3389/fpsyt.2022.853428

**Published:** 2022-04-26

**Authors:** Xiaoyu Fu, Wenxiang Quan, Lijun Liu, Tian Li, Wentian Dong, Jiuju Wang, Ju Tian, Jun Yan, Jinmin Liao

**Affiliations:** ^1^Peking University Sixth Hospital, Peking University Institute of Mental Health, NHC Key Laboratory of Mental Health (Peking University), National Clinical Research Center for Mental Disorders (Peking University Sixth Hospital), Beijing, China; ^2^Zhongshan Hospital, Fudan University, Xiamen, China

**Keywords:** obsessive-compulsive disorder, schizophrenia, near-infrared spectroscopy (NIRS), verbal fluency task (VFT), brain activation

## Abstract

Schizophrenia (SZ) and obsessive-compulsive disorder (OCD) share several epidemiological and clinical features, but the neurobiological substrates shared by these two diseases remain unclear. This study aimed to explore the similarities and differences in brain function between them using near-infrared spectroscopy (NIRS). Eventually, 130 SZ patients, 70 OCD and 75 normal controls (NCs) were enrolled. A 52-channel NIRS instrument was used to detect the concentration changes in oxygenated hemoglobin ([oxy-Hb]) during the verbal fluency task. Ten regions of interests (ROIs) were defined: the bilateral dorsolateral prefrontal cortex (DLPFC), frontopolar cortex (FPC), orbitofrontal cortex (OFC), inferior prefrontal gyrus (IFG) and temporal gyrus (TG). Through two different analysis strategies based on channels or ROIs, we compared the [oxy-Hb] changes in three groups by one-way analysis of variance (ANOVA) and *post-hoc* tests. Across 52 channels, compared to the NC group, both SZ and OCD groups exhibited reduced activity in 17 channels, including left FPC, left DLPFC, bilateral OFC, IFG, middle TG, supplementary motor cortex and Broca’s area, while SZ showed lower activity in channel 35 (right OFC) than OCD patients. Across all ROIs, compared to the NC group, both SZ and OCD groups showed reduced activity in 7 ROIs, including left FPC, bilateral OFC, IFG and TG, while SZ showed lower activity in the right OFC than OCD group, which were almost consistent with the results based on channels. This study suggests SZ and OCD present with some similar neuropathological changes, while SZ shows more severe impairment in the right OFC than OCD.

## Introduction

Schizophrenia (SZ) and obsessive-compulsive disorder (OCD) are two chronic and debilitating mental disorders. Although they are two distinct disorders, they have similar clinical symptoms, influences on cognitive function and epidemiological trends. Regarding psychopathology, some researchers have suggested that delusions or hallucinations and obsessions might coexist in a unique psychopathologic complex, and a key similarity is related to the “need to control thoughts” (1, 2). Hallucinations and delusions in SZ and obsessive thoughts in OCD are both unwanted and involve intrusive mental activities. Both groups worry about these thoughts and feel out of control. SZ patients attribute troubling thoughts or voices to other persons, leading to abnormal behaviors. In OCD patients, obsessions trigger compulsions and avoidances aimed at neutralizing worries. In addition, SZ and OCD both show cognitive impairment in many domains, including memory, executive function, goal-directed behavior, response inhibition, source-monitoring and social cognition, and SZ has greater deficits than OCD (1, 3–7). Furthermore, there are increasing epidemiological studies showing the relationships between the two disorders. OCD-symptoms may appear at different stages of SZ: prodromal symptoms, co-occurrence of OCD, SZ and antipsychotic-induced OCD (8). The prevalence of comorbidity in SZ and OCD may range between 5 and 23% (8–10). Three national cohort studies attempted to weigh the association of baseline OCD diagnosis and the risk of SZ, and they discovered that patients with OCD had a greater risk of schizophrenia over time than the general population (11–13). Cheng et al. investigated 2009 patients who were first diagnosed with OCD from 2000 to 2013 and discovered that the OCD group had a significantly greater risk of schizophrenia (hazard ratio = 30.29, 95% confidence interval (CI) = 17.91–51.21) (13). These studies demonstrate the clinical and phenomenological relevance of SZ and OCD. However, little is known about the neurobiology. Therefore, it is important to explore the neurobiological substrates and the etiological relationship between SZ and OCD.

The neurobiological substrates of both SZ and OCD are thought to be related to neurodevelopment (14). Brain structure and function are thought to reflect common trajectories in brain development and maturation. Neuroimaging studies have provided clues to the pathophysiology of these two disorders. Few studies have directly compared OCD with SZ from neuroimaging aspects. In functional magnetic resonance imaging (fMRI) studies (15), compared with healthy controls, researchers found that both SZ and OCD patients showed increased resting-state functional connectivity between subregions of the default network (DMN) and the executive control network (ECN). In addition, the connection between the DMN and the middle temporal gyrus increased in the SZ group, while the connection was reduced in the OCD group. In a study of unmedicated patients with SZ or OCD (16), similarly areas of functional abnormality were highlighted, such as frontal, temporal, occipital, and subcortical regions. Furthermore, the white matter fiber connections were more severely damaged in SZ. Previous MRI studies have shown that SZ and OCD share common areas of functional abnormality, and the abnormality is more pronounced in schizophrenia.

Multichannel near-infrared spectroscopy (NIRS) is another promising and non-invasive optical imaging technique to measure concentration changes of oxygenated hemoglobin ([oxy-Hb]), deoxygenated hemoglobin ([deoxy-Hb]), and total hemoglobin ([total- Hb]) related to functional activity in the brain (17) (18). Furthermore, because of its low maintenance cost, portability, and tolerance to micromotion, NIRS has been commonly used to determine brain function in psychiatric patients. The verbal fluency task (VFT) is one of the most commonly used methods for measuring cognitive activation in NIRS. During the VFT task, the prefrontal and temporal lobes were activated.

Previous studies have shown that patients with SZ had lower activation of the prefrontal cortex (PFC) and temporal cortex during VFT. Takizawa et al. (19) used 52-channel NIRS and found reduced activity in bilateral frontal pole areas in 55 patients with SZ, while Shimodera et al. (20) used 42-channel NIRS and found reduced activity in bilateral ventral lateral PFC regions in 31 patients with SZ. The above inconsistent results may be related to the small sample size and NIRS channel numbers. Quan et al. (21) used 52-channel NIRS on 140 patients with schizophrenia and found that in addition to the PFC, the temporal cortex was also underactivated. NIRS studies of OCD showed that the prefrontal and temporal cortices had decreased hemodynamic responses during VFT. Reduced [oxy-Hb] changes in the right dorsolateral prefrontal cortex (DLPFC) were discovered when using 42-channel NIRS for VFT in 20 patients with OCD (22). When a 52-channel NIRS was used in 70 patients with OCD, we found that decreased hemodynamic responses in the prefrontal and temporal cortex, as well as abnormal brain activation, were linked to the severity of OCD symptoms (23). To the best of our knowledge, the NIRS studies applied to the either SZ or OCD suggested decreased activation in the PFC and temporal cortex, however, no direct comparison between them had been reported.

Therefore, in this study, we aimed to explore the similarities and differences in brain function between the two disorders by using a 52-channel NIRS system to examine the brain activation characteristics in SZ and OCD patients under VFT. We proposed the hypothesis that OCD or SZ patients presented with some homologous brain region abnormalities when compared to normal controls (NCs), while patients in the SZ group might suffer from more severe degree of brain impairment than those in the OCD group.

## Materials and Methods

### Participants

The patients with SZ or OCD were recruited from the inpatient and outpatient services at Peking University Sixth Hospital. Two experienced psychiatrists independently diagnosed each patient using the Structured Clinical Interview for DSM-IV-TR Axis I Disorders (SCID-I, patient edition). The following requirements were required for enrollment: (a) age between 16 and 55; (b) right-handedness; and (c) accomplished the primary education (more than 6 years of education). Patients with other mental disorders, neurological disease, brain injury, or a severe medical condition were excluded, as were patients who had received electroconvulsive therapy within the previous 6 months. Handedness was assessed by Edinburgh Handedness Questionnaire (24). This study was based on our previous study, and the OCD patients in this study overlapped in our previous study (23). Our team’s previous article (23) can be referenced for specific OCD sample information. Normal subjects were recruited from the local community, and they were required to meet the same inclusion criteria as the patient sample, but they also had to be free of any prior or current psychiatric disorders, who were screened by using the SCID-I (non-patient edition). This study was approved by the Ethics and Research Committees of Peking University Sixth Hospital (Institute of Mental Health) [(2014), No. 24]. After learning about the experimental process, all of the subjects willingly signed an informed consent form.

### Task Description

**FIGURE 1:**
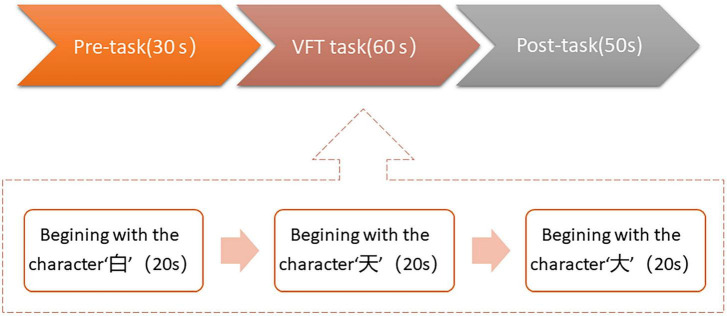
Overall verbal fluency task (VFT) task flow in this study. The task was divided into three periods: a 30 s pre-task baseline period, a 60s VFT task period, and a 50 s post-task baseline period.

We used the Chinese version of the VFT in this study, which was created for Chinese participants by Quan et al. (21). In Figure 1, the test is divided into three sections: a 30-s pretask baseline period, a 60-s task period and a 50-s posttask baseline period. During the 60-s task period, three Chinese characters were shown on the screen: “白” (which indicates white), “天” (which indicates sky), and “大” (which indicates big), and the time for each word group was limited to 20 s. During the task period, the subjects were required to verbally produce as many phrases as they could, starting with each given character. During VFT, the assessment of cognitive function was based on the total number of correct phrases in each of the three groups.

### Near-Infrared Spectroscopy Measurements

We used a 52-channel NIRS optical terrain system (ETG-4000, Hitachi Medical Corporation, Tokyo, Japan) with two near-infrared light wavelengths to collect data (695 and 830 nm). According to the modified Beer-Lambert law, the system measured the relative concentrations of [oxy-Hb], [deoxy-Hb], and [total-Hb] changes (25). The instrument consisted of 17 light emitters and 16 light detectors grouped in a 3 × 11 array with a 3.0 cm distance between probes, which was the same as the previous studies (26). According to the international 10–20 system, the lowest probes were positioned along the Fp1-Fp2 line, as in a previous study (19). We determined the brain region of each NIRS channel based on the mapping between Brodmann’s areas and the 10–20 electrode positions (27). The probes were arranged to measure [oxy-Hb] and [deoxy-Hb] relative concentration changes in the bilateral frontal and temporal cortices (including Brodmann’s areas (BA) 2, 6, 9, 10, 11, 21, 22, 40, 43, 45, 46, and 47). The time resolution of NIRS absorption was set to 0.1 s.

The statistical analyses of NIRS data were performed using the Statistical Package for Social Sciences (SPSS) version 24 (IBM Corporation, New York, NY, United States), NIRS-SPM^1^ and MATLAB 2013b. As changes in [oxy-Hb] had been proved to be the most sensitive indicator of local blood flow in the brain in animal studies (28), we focused our research on [oxy-Hb] concentration changes, and we provided the results of [deoxy-Hb] concentration changes in the supplementary. The main steps and parameters used for the analysis in this study can refer to our previous study (23). To make our findings more robust, we tried two different analysis strategies by each channels and regions of interest (ROIs) to compare the [oxy-Hb] changes during the VFT (29).

### Regions of Interest

**FIGURE 2:**
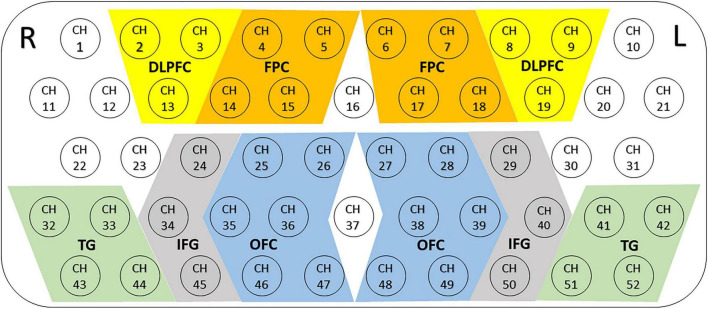
Locations and definitions of the 10 regions of interest (ROIs). The 52 measuring areas are labeled as ch1 to ch52 from the right posterior to left anterior. Ten ROIs consisted of the bilateral dorsolateral prefrontal cortex (DLPFC), the bilateral frontopolar cortex (FPC), the bilateral orbitofrontal cortex (OFC), the bilateral inferior prefrontal gyrus (IFG) and temporal gyrus (TG); R, right; L, left.

We defined 10 ROIs, which covered the main bilateral frontal and temporal cortices (see Figure 2): bilateral dorsolateral prefrontal cortex (DLPFC), frontopolar cortex (FPC), orbitofrontal cortex (OFC), inferior prefrontal gyrus (IFG) and temporal gyrus (TG). The results show that the right DLPFC region was defined as that covered by channels 2–3 and 13; the left DLPFC region was defined as that covered by channels 8–9 and 19; the right FPC region was defined as that covered by channels 4–5 and 14–15; the left FPC region was defined as that covered by channels 6–7 and 17–18; the right OFC region was defined as that covered by channels 25–26, 35–36, and 46–47; the left OFC region was defined as that covered by channels 27–28, 38–39, and 48–49; the right IFG region was defined as that covered by channels 24, 34 and 45; the left IFG region was defined as that covered by channels 29, 40, and 50; the right TG region was defined as that covered by channels 32–33 and 43–44; the left TG regions was defined as that covered by channels 41–42 and 51–52. The averaged values of mean [oxy-Hb] changes in each ROI were measured for data analysis.

### Statistical Analyses

The chi-square test was used to compare categorical variables. The normally distributed clinical variables were analyzed using one-way analysis of variance (ANOVA), while Kruskal–Wallis (H) test was used on the non-normally distributed variables. We performed the normality test of datasets by Shapiro-Wilk test and found the histograms of either total samples or subgroup samples of [oxy-Hb] changes were approximate normality. Thus, we used one-way ANOVA for each channel to compare [oxy-Hb] changes in patients with SZ, OCD and NC during the task period. Then, *post-hoc* pairwise comparisons were used to locate differences in the activation time of each brain region between patients with SZ, OCD, and NC during the task period, and the result was significant using a Bonferroni-corrected alpha level of 0.0167 (0.05/3). We set the *q*-value specifying the maximum false discovery rate (FDR) to 0.05, so that the average false-positive rate was no greater than 5% during the processing of [oxy-Hb] data obtained from multiple channels (30).

## Results

### Demographic Data and Clinical Characteristics

Demographic and clinical data are shown in Table 1. Study participants included 130 patients with SZ (male/female: 67/63, age: mean 27.8 ± 8.4 years), 70 patients with OCD (male/female: 44/26, age: mean 27.7 ± 9.8 years), and 75 NCs (male/female: 50/25, age: mean 29.9 ± 8.0 years). There were no significant differences in age [*F* (2,275) = 1.949, *p* = 0.144], sex [Chi-square test: χ^2^ (2) = 5.204, *p* = 0.074], or education level [*F* (2, 275) = 1.341, *p* = 0.263] between the three groups. There was also no significant differences in duration of illness [*t* = 1.313, *p* = 0.191] between the SZ and OCD groups. Patients with SZ were treated with antipsychotics, and 92 SZ patients received single antipsychotic and 38 SZ patients received combined antipsychotic treatment, and the chlorpromazine equivalent doses ranged from 56 to 1 575 mg daily (mean dose: 470.43 ± 264.01 mg/day). Thirty-four patients with OCD were unmedicated, and 36 patients with OCD only received antidepressants, who did not receive anti-psychotics. More medication details are shown in supplementary.

**TABLE 1:** Sample characteristics and verbal fluency task (VFT) performance of patients and normal controls.

	SZ group (*n* = 130)	OCD group (*n* = 70)	NC group (*n* = 75)	F/χ ^2^/t Value	P ^b^ Value	Significance^c^
Age(year)	27.8 ± 8.4	27.7 ± 9.8	29.9 ± 8.0	1.949	0.144	
Gender(M/F)^a^	67/63	44/26	50/25	5.204	0.074	
Education(year)	14.3 ± 2.8	14.3 ± 2.3	14.9 ± 3.0	1.341	0.263	
Duration of illness(year)	6.32 ± 6.70	4.97 ± 5.77		1.313	0.191	
VFT^c^	9.7 ± 4.2	10.5 ± 4.0	11.2 ± 4.2	3.174	0.044	SZ vs. NC *p* = 0.042 OCD vs. NC *p* = 1.000 SZ vs. OCD *p* = 0.639

*NC, normal controls; SZ, schizophrenia; OCD, obsessive-compulsive disorder; VFT, Verbal Fluency Test.*

*^a^ M for male and F for female.*

*^b^ χ^2^ test for gender, one-way ANOVAs for age, education and VFT, t-test for the duration of illness.*

*^c^ Significant group differences are shown to the right. p < 0.05 (Bonferonni correction) was considered significant.*

### Comparisons of Verbal Fluency Task Performance Among Three Groups

There were significant between-group differences in the VFT score (*F* = 3.174, *p* = 0.044). The effect size of the ANOVA of VFT score among three groups was 0.024, which was small. *Post-hoc* pairwise comparisons revealed that the SZ group had lower VFT scores than the NC group (NC: 11.2 ± 4.2, SZ: 9.7 ± 4.2; *p* = 0.042), but there was no significant difference between the OCD and NC groups (NC: 11.2 ± 4.2, OCD: 10.5 ± 4.0; *p* = 1.000) and between the SZ and OCD groups (*p* = 0.639).

### Comparisons of [oxy-Hb] Changes Among Three Groups by Each Channel

**FIGURE 3:**
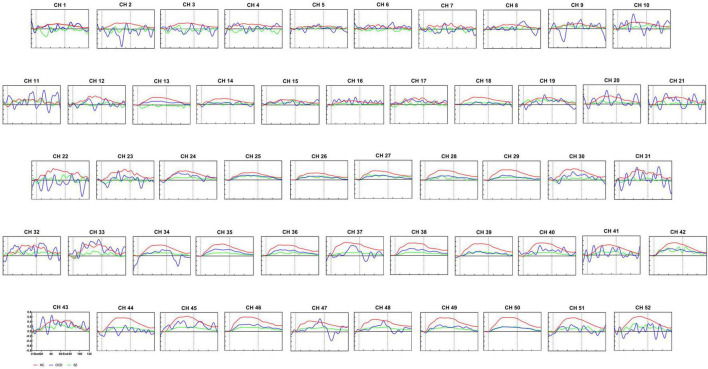
Waveforms of [oxy-Hb] changes during the verbal fluency task. Grand average waveforms for each of the 52 channels revealing alterations in [oxy-Hb] concentration during VFT in the normal controls group (red line), the OCD group (blue line) and schizophrenia group (green line). The time course(s) is shown on the x-axis, and the change in [oxy-Hb] concentration is shown on the y-axis (mM.cm). The pre-task baseline was set at 10 s, the task timeframe was set at 10–70 s, and the post-task period was set at 50 s. The vertical lines mark the beginning and end of VFT. NC, normal controls; SZ, schizophrenia; OCD, obsessive-compulsive disorder.

**FIGURE 4:**
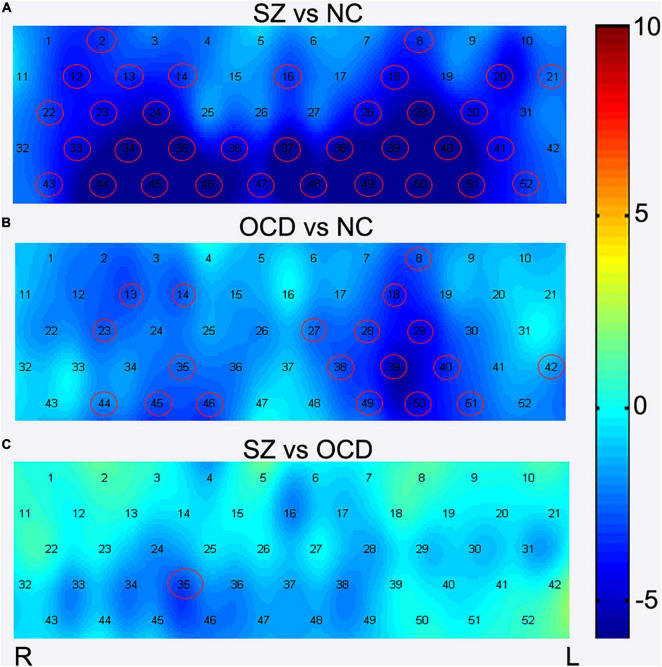
Comparisons of [oxy-Hb] changes among three groups by each channel. During the VFT, brain activation was measured by alterations in [oxy-Hb] concentrations in patients with OCD, SZ, and NC. The channels that reached statistical significance were represented by red cycles. *p* < 0.05, FDR correction. Color bar represent *t* values. **(A)** The group comparison for brain activation between the SZ patients and NC. **(B)** The group comparison for brain activation between the OCD patients and NC. **(C)** The group comparison for brain activation between the SZ and OCD patients. SZ, schizophrenia NC, normal controls; OCD, obsessive-compulsive disorder; R, right; L, left.

Among the patients with OCD, SZ and NCs, after multiple comparison corrections (*p* < 0.05, FDR corrected), we found substantial primary group effects for [oxy-Hb] changes in the 35 channels (Ch. 2, 8, 12–14, 16, 18, 20–21, 23–24, 27–30, and 33–52) by ANOVA. Figure 3 shows grand-averaged waveforms of [oxy-Hb] changes during VFT in 52 NIRS channels between the three groups. In addition, the results of *post-hoc* tests demonstrated that the SZ group had significantly lower [oxy-Hb] concentration changes than the NC group in 34 channels (Ch. 2, 8, 12–14, 16, 18, 20–24, 28–30, 33–41, 43–52) (Figure 4A). The 34 channels comprised the following areas of the brain: bilateral premotor and supplementary motor cortex (SMA) (BA6, Ch. 2, 12, 20, 23), bilateral FPC(BA10, Ch. 14, 16, 18), bilateral OFC (BA11, Ch. 28, 35–39, 46–50), bilateral middle temporal gyrus (MTG) (BA21, Ch. 44, 51, 52), bilateral superior temporal gyrus (STG) (BA22, Ch. 33, 41, 43), bilateral supramarginal gyrus part of Wernicke’s area (BA40, Ch. 21, 22), right pars triangularis Broca’s area (BA45, Ch. 13, 30), left DLPFC (BA 46, Ch. 8), and bilateral IFG (BA47, Ch. 24, 29, 34, 40, 45). The uncorrected results were shown in Supplementary Table 1.

We also found that the concentration in the task-induced [oxy-Hb] changes were markedly lower in the patients with OCD than in the NCs in 19 channels (Ch. 8, 13–14, 18, 23, 27–29, 35, 38–40, 42, 44–46, 49–51) (Figure 4B). The 19 channels comprised the following areas of the brain: SMA (BA6, Ch. 23), bilateral FPC (BA10, Ch. 14, 18), bilateral OFC (BA11, Ch. 27, 28, 35, 38, 39, 46, 49, 50), bilateral MTG (BA21, Ch. 44,51), left STG (BA22, Ch. 42), right pars triangularis Broca’s area (BA45, Ch. 13), left DLPFC (BA 46, Ch. 8), and bilateral IFG (BA47, Ch. 29, 40, 45). All results were corrected using the FDR correction. The uncorrected results were shown in Supplementary Table 2.

In summary, there are 17 common channels that have lower cortical activation in both the patients with OCD and those with SZ, including bilateral FPC (BA 10, CH 14,18), bilateral OFC (BA11, CH. 28, 35, 38–39, 46, 49–50), left DLPFC (BA46,CH. 8), bilateral IFG (BA47, CH 29,40,45), right SMA (BA 6, CH. 23), bilateral MTG (BA21, CH. 44,51) and right pars triangularis Broca’s area (BA45, CH. 13).

In addition, after FDR correction (*p* < 0.05), the results of *post-hoc* tests showed that the SZ group had lower [oxy-Hb] concentration in channel 35 than the OCD group (see Figure 3 and Figure 4C). Channel 35 represented the brain area of the right OFC (BA 11, MNI coordinates [48 52 −13]).

### Comparisons of [oxy-Hb] Changes Among Three Group by Regions of Interests

**FIGURE 5:**
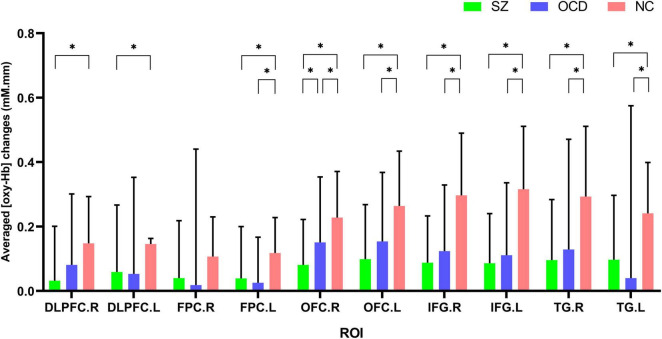
Comparisons of [oxy-Hb] changes by regions of interest (ROIs) among the SZ group (green color), the OCD group (blue color) and the NC group (red color). NC, normal controls; SZ, schizophrenia; OCD, obsessive-compulsive disorder; DLPFC, dorsolateral prefrontal cortex; FPC, frontopolar cortex; OFC, orbitofrontal cortex; IFG, inferior prefrontal gyrus; TG, temporal gyrus. R, right; L, left. *p < 0.05, FDR corrected.

We further performed comparisons of brain activation among the three groups according to ROIs, and the results showed that significant group effects between SZ, OCD and NCs in nine ROIs (see Figure 5), including the bilateral DLPFC, OFC, IFG, TG and left FPC. Further, *post-hoc* test results revealed that the SZ group had significantly lower [oxy-Hb] concentration changes in nine regions (bilateral DLPFC, OFC, IFG, TG and left FPC) than the NC group, while the OCD group had significantly lower [oxy-Hb] concentration changes in seven regions (bilateral OFC, IFG, TG and left FPC) than the NC group. We also found that right OFC had significantly lower concentration changes in SZ group than OCD group during the VFT. All results were corrected using the FDR correction, *p* < 0.05.

## Discussion

To the best of our knowledge, this is the first NIRS study that directly compares OCD and SZ. Compared to the NC group, both SZ and OCD groups showed decreased activity in the left FPC, left DLPFC, bilateral OFC, bilateral IFG, bilateral TG, right SMA and right Broca’s area. Additionally, compared to the OCD group, the SZ group had more pronounced reduced activity in the right OFC. The main results analyzed by ROI were almost consistent with the findings based on channels analysis, thus we consider those findings are robust to some extent. These results support the hypothesis that the two disorders have partially similar brain functional imaging changes, and SZ has more serious impairment, which helps us to better understand the correlations and discrepancies between the two disorders in phenomenology.

Patients with SZ and OCD both presented reduced activity in brain regions of DLPFC, FPC, OFC, IFG, MTG, SMA and Broca’s area. This finding was consistent with prior research. Previous NIRS studies in either disorders also found reduced activation of the prefrontal and temporal lobes (19, 21–23). The two disorders shared some similar functional impaired brain areas, which may improve understanding of the phenomenological, clinical, and cognitive associations between SZ and OCD. The similar impairment pattern of brain function in SZ and OCD may be the interpretation of phenomenological overlap, including a high prevalence of comorbidities, a wide range of schizo-obsessive spectrum disorders and risk factors for each other (8–10). And the comorbidity of the two disorders would cause more neurocognitive impairment, poorer quality of life, and treatment resistance (31).

The shared changes in brain function in SZ and OCD were consistent with the overlapping cognition deficits. SZ and OCD patients presented with homologous impaired cognition in memory, executive function, goal-directed behavior, response inhibition, source monitoring and social cognition (3–7, 32). The DLPFC is crucial for working memory and episodic memory processes (33, 34). A study on working memory in SZ and OCD patients using the Wechsler Memory Inventory (32) found that both groups had worse visual memory function than normal controls. This study found that both SZ and OCD manifested reduced activity in the DLPFC, suggesting that impaired function of the DLPFC might be the neural basis of memory deficits in SZ and OCD. The OFC is a key hub on the expansive neural circuitry of sociality (35). Some studies found that both SZ and OCD showed poor social cognition, and both had impairments in emotion recognition and theory of mind (7). Furthermore, the OFC, SMA and IFG are generally considered to be related to behavior inhibition (36, 37). Previous studies had investigated response inhibition in SZ and OCD by stopping signal task and found impaired response inhibition in both disorders (5). Combined with clinical findings, inhibition difficulties are evident in OCD patients, who are unable to stop their obsessions and compulsions and repeatedly engage in compulsive behaviors, while patients with SZ are impaired in behavior regulation, with frequent aggression and impulsivity. These neuroimaging impairments are relevant for understanding clinical symptoms in SZ and OCD.

Furthermore, our study also found that the SZ group showed more serious impairment in the right OFC than the OCD group, which is consistent in two different analysis methods by each channel or ROIs. This finding is consistent with prior research by MRI. Qin et al. found decreased gray matter volume in the OFC of schizophrenia patients with no significant changes found in OCD patients, and damage to white matter structure was more severe in SZ patients than in OCD patients (16). The OFC plays a key role in executive function, behavioral flexibility, social cognition and insight (38–40) and is involved in the pathogenesis of SZ (41, 42) and OCD (39, 43, 44). A large body of evidence suggests that executive dysfunction is a core feature of both schizophrenia (45) and OCD patients (46). Our results can be interpreted as the patients with schizophrenia might have more executive dysfunction than OCD patients. Resting-state fMRI research found that the functional connectivity between the right anterior insula and right OFC correlated with insight level in OCD patients, perhaps as a result of poor encoding and integration of self-evaluative data on beliefs and behaviors (40). Approximately 22–25% of patients with OCD have poor insight, meaning they are unable to accept the excessiveness or unreasonableness of their actions (47), while the prevalence of poor insight in SZ was 43.4% in a meta-analysis (48). Cognitive deficits of source monitoring are involved in the process of poor sight. A study found that both SZ and OCD showed impaired internal source-monitoring abilities, and compared to patients with OCD, patients with SZ had abnormal reality monitoring. These patients all have difficulty tracking their own minds, which causes them to be confused about what they actually did or interpreted and what they envisioned (6). In conclusion, the results we obtained by NIRS may help us better understand the characteristics of cognitive deficits and insight in SZ and OCD.

There are some limitations to our study. First, according to some precious NIRS studies in SZ and OCD at both early stage or chronic stage, no correlations between antipsychotic dosage and NIRS signals were found (49, 50). We did not plan to collect information about duration of antipsychotics/antidepressants usage in the initial designing scheme. Further studies enrolling untreated SZ and OCD patients may be needed to evaluate the potential effect. Second, according to the retrospective nature of this study, we did not assess severity of the clinic symptoms of patients with SZ and OCD. However, two professional psychiatrists had already confirmed that the participants were free of psychiatric comorbidities. Besides, as we enrolled consecutive participants consented to engage in this research from outpatients and inpatients, thus we considered the results could, to some extent, represent the characteristics of general patient populations.

## Conclusion

In summary, this study investigated the similarities and differences of brain function in SZ and OCD by NIRS through two different analysis strategies based on channels or ROIs. We have found that the two patient groups have partially similar neuropathological changes, and SZ has more severe impairment in the right OFC than OCD patients. The similar disrupted pattern of brain function in both clinical groups will help us understand the associations of clinical symptoms, cognitive function and epidemiological trends, and the differences in brain function will improve understanding of the distinctions between SZ and OCD.

## Data Availability Statement

The datasets presented in this article are not readily available because they contain potentially sensitive patient information. Requests to access the datasets should be directed to JL, jinminliao@bjmu.edu.cn.

## Ethics Statement

The studies involving human participants were reviewed and approved by the Ethics and Research Committees of Peking University Sixth Hospital (Institute of Mental Health) [(2014), No. 24]. The patients/participants provided their written informed consent to participate in this study. Written informed consent was obtained from the individual(s) for the publication of any potentially identifiable images or data included in this article.

## Author Contributions

XF and WQ contributed to the study design, statistical analyses, and writing of the manuscript. LL, TL, WD, JW, and JT contributed to the data acquisitions, data management, and writing of the manuscript. JY contributed to the original study design, statistical analyses, critical reading, and writing of the manuscript. JL contributed to the data management, statistical analyses, critical reading, and writing of the manuscript. All authors reviewed the article and provided the approval for publication.

## Conflict of Interest

The authors declare that the research was conducted in the absence of any commercial or financial relationships that could be construed as a potential conflict of interest.

## Publisher’s Note

All claims expressed in this article are solely those of the authors and do not necessarily represent those of their affiliated organizations, or those of the publisher, the editors and the reviewers. Any product that may be evaluated in this article, or claim that may be made by its manufacturer, is not guaranteed or endorsed by the publisher.

## Footnotes

1
https://www.nitrc.org/projects/nirs_spm/
