# Supplementary material for: Similarities and Differences in Brain Activation Between Patients With Schizophrenia and Obsessive-Compulsive Disorder: A Near-Infrared Spectroscopy Study
Source: Front Psychiatry. 2022 Apr 26;13:853428. doi: 10.3389/fpsyt.2022.853428 (PMC9086627; doi:10.3389/fpsyt.2022.853428)
Supplement: Supplementary file 1 [file Data_Sheet_1.pdf]

Supplementary material

Similarities and Differences in Brain Activation between Patients with Schizophrenia and Obsessive-Compulsive Disorder: A Near-Infrared Spectroscopy Study

1. Medication information for patients with schizophrenia and obsessive-compulsive disorder

In our study, 130 patients with schizophrenia (SZ) and seventy patients with obsessive-compulsive disorder (OCD) were involved in the analysis of results. 130 patients with schizophrenia received antipsychotics, as follows: 24 patients received risperidone, 33 patients received olanzapine, 5 patients received quetiapine, 3 patients received aripiprazole, 11 patients received clozapine, 3 patients received ziprasidone, and 28 patients received paliperidone, 1 patients received sulpiride, 10 patients received amisulpride, 2 patients received perphenazine, 3 patients received chlorpromazine, 3 patients received haloperidol, 2 patients received zolidine, 1 patients received branseline. Among them, 38 patients were co-medicated, and the dose of all schizophrenia patients after conversion to chlorpromazine was 56-1575 mg/d (mean dose: 470.43 ± 264.01 mg/day).

Thirty-four patients with OCD were unmedicated, and 36 patients with OCD received antidepressants, as follows: 15 patients received sertraline, 12 patients received fluvoxamine, 5 patients received paroxetine, 2 patients received escitalopram, 2 patients received fluoxetine, 2 patients received clomipramine, and 2 patients received venlafaxine.

2. The uncorrected results of comparisons of [oxy-Hb] changes among the patients with OCD, SZ and NC.

Table S1.The channels of significantly different brain activation of [oxy-Hb] during VFT between SZ and NC group.

Channel	Brain area	BA	MINI coordinates			T	*p*
			X	Y	Z		
1	Supramarginal gyrus part of Wernicke's area(R)	40	56	-28	57	-2.233	0.027
2	Anterior motor cortex(R)	6	59	2	47	-3.447	0.001*
4	Frontopolar area(R)	10	39	60	17	-2.369	0.019
8	Dorsolateral prefrontal cortex	46	-46	42	27	-2.956	0.003*
12	Anterior motor cortex(R)	6	65	-12	42	-3.968	<0.001*
13	Pars triangularis Broca's area(R)	45	60	24	23	-3.273	0.001*
14	Frontopolar area(R)	10	49	52	10	-3.354	0.001*

*, p <0.05, FDR corrected

Continued from the Table S1

Channel	Brain area	BA	MINI coordinates			T	p
			X	Y	Z		
16	Frontopolar area(L)	10	12	73	-4	-3.238	0.001*
17	Frontopolar area(L)	10	-17	72	-4	-2.037	0.043
18	Frontopolar area(L)	10	-41	60	1	-4.386	<0.001*
19	Dorsolateral prefrontal cortex(L)	46	-55	34	15	-2.111	0.036
20	anterior motor cortex(L)	6	-65	-6	34	-4.756	<0.001*
21	Supramarginal gyrus part of Wernicke's area(L)	40	-63	-37	48	-2.442	0.015*
22	Supramarginal gyrus part of Wernicke's area(R)	40	69	-28	40	-2.799	0.006*
23	anterior motor cortex(R)	6	66	8	19	-4.329	<0.001*
24	inferior prefrontal cortex(R)	47	56	42	0	-5.313	<0.001*
26	Orbitofrontal area(R)	11	19	69	-15	-2.177	0.031
27	Orbitofrontal area(L)	11	-1	68	-15	-2.355	0.019
28	orbitofrontal cortex(L)	11	-26	66	-13	-4.677	<0.001*
29	inferior prefrontal cortex(L)	47	-49	49	-9	-6.206	<0.001*
30	pars triangularis Broca's area(L)	45	-60	17	9	-4.157	<0.001*
31	Subcentral area(L)	43	-68	-17	26	-2.274	0.024
32	Supramarginal gyrus part of Wernicke's area(R)	1	68	-40	34	-2.202	0.029
33	superior temporal gyrus(R)	22	70	-10	11	-4.508	<0.001*
34	inferior prefrontal cortex(R)	47	58	32	-2	-6.645	<0.001*
35	orbitofrontal cortex(R)	11	48	52	-13	-7.429	<0.001*
36	orbitofrontal cortex(R)	11	30	66	-14	-5.391	<0.001*
37	orbitofrontal cortex(R)	11	9	68	-20	-7.214	<0.001*
38	orbitofrontal cortex(L)	11	-11	66	-20	-6.537	<0.001*
39	orbitofrontal cortex(L)	11	-37	58	-14	-7.355	<0.001*
40	inferior prefrontal cortex(L)	47	-52	40	-11	-7.407	<0.001*
41	superior temporal gyrus(L)	22	-64	-1	-1	-3.808	<0.001*
42	Superior Temporal Gyrus(L)	22	-69	-34	17	-2.215	0.028
43	superior temporal gyrus(R)	22	73	-26	2	-2.629	0.009*
44	Middle Temporal gyrus(R)	21	64	5	-17	-7.473	<0.001*
45	inferior prefrontal cortex(R)	47	49	46	-17	-8.533	<0.001*
46	orbitofrontal cortex(R)	11	38	58	-17	-6.862	<0.001*
47	orbitofrontal cortex(R)	11	14	65	-22	-4.488	<0.001*
48	orbitofrontal cortex(L)	11	1	62	-24	-5.300	<0.001*
49	orbitofrontal cortex(L)	11	-19	61	-19	-7.204	<0.001*
50	orbitofrontal cortex(L)	11	-45	45	-18	-7.609	<0.001*
51	middle temporal gyrus(L)	21	-58	11	-26	-4.767	<0.001*
52	middle temporal gyrus(L)	21	-70	-15	-14	-2.947	0.004*

*, p <0.05, FDR corrected

Table S2. The channels of significantly different brain activation of [oxy-Hb] during VFT between OCD group and NC group.

Channel	Brain area	BA	MINI coordinates			T	P
			X	Y	Z		
2	Anterior motor cortex (R)	6	59	2	47	-2.296	0.023
8	dorsolateral prefrontal cortex(L)	46	-46	42	27	-2.452	0.015*
12	Anterior motor cortex (R)	6	65	-12	42	-2.330	0.021
13	pars triangularis Broca's area(R)	45	60	24	23	-3.159	0.002*
14	Frontopolar area(R)	10	49	52	10	-2.834	0.005*
18	Frontopolar area(L)	10	-41	60	1	-3.793	<0.001*
22	Supramarginal gyrus part of Wernicke's area(R)	40	69	-28	40	-1.978	0.049
23	anterior motor cortex(R)	6	66	8	19	-2.713	0.007*
24	Inferior prefrontal gyrus(R)	47	56	42	0	-2.153	0.033
26	Orbitofrontal area(R)	11	19	69	-15	-2.013	0.046
27	orbitofrontal cortex(L)	11	-1	68	-15	-2.655	0.009*
28	orbitofrontal cortex(L)	11	-26	66	-13	-3.282	0.001*
29	inferior prefrontal cortex(L)	47	-49	49	-9	-4.274	<0.001*
30	pars triangularis Broca's area(L)	45	-60	17	9	-2.086	0.039
35	orbitofrontal cortex(R)	11	48	52	-13	-2.651	0.009*
36	Orbitofrontal area(R)	11	30	66	-14	-2.270	0.025
38	orbitofrontal cortex(L)	11	-11	66	-20	-3.088	0.002*
39	orbitofrontal cortex(L)	11	-37	58	-14	-5.696	<0.001*
40	inferior prefrontal cortex(L)	47	-52	40	-11	-3.267	0.001*
42	superior temporal gyrus(L)	22	-69	-34	17	-2.488	0.014*
44	middle temporal gyru(R)	21	64	5	-17	-2.620	0.010*
45	inferior prefrontal cortex(R)	47	49	46	-17	-3.417	0.001*
46	orbitofrontal cortex(R)	11	38	58	-17	-3.396	0.001*
49	orbitofrontal cortex(L)	11	-19	61	-19	-2.599	0.010*
50	orbitofrontal cortex(L)	11	-45	45	-18	-4.256	<0.001*
51	middle temporal gyrus(L)	21	-58	11	-26	-2.807	0.006*
52	middle Temporal gyrus(L)	21	-70	-15	-14	-2.130	0.035

*, p<0.05, FDR corrected.

Table S3. The channels of significantly different brain activation of [oxy-Hb] during VFT between SZ group and OCD group.

Channel	Brain area	BA	MINI coordinates			T	P
			X	Y	Z		
16	Frontopolar area(L)	10	12	73	-4	-2.197	0.029
33	superior temporal gyrus(R)	22	70	-10	11	-2.118	0.035
34	Inferior prefrontal gyrus(R)	47	58	32	-2	-2.348	0.020
35	orbitofrontal cortex(R)	11	48	52	-13	-3.351	<0.001*
36	orbitofrontal area(R)	11	30	66	-14	-2.152	0.033
38	orbitofrontal cortex(L)	11	-11	66	-20	-2.208	0.028
42	superior temporal gyrus(L)	22	-69	-34	17	2.185	0.030
46	orbitofrontal cortex(R)	11	38	58	-17	-2.254	0.025

*, $p<0.05$, FDR corrected.

3. Comparisons of brain activation of [deoxy-Hb] among the patients with OCD, SZ and NC (see Fig. S1).

Among the patients with OCD, SZ and NC, we found substantial primary group effects for [deoxy-Hb] concentration changes in 17 channels ($p < 0.05$, FDR corrected) by ANOVA , including Ch. 5, 23, 28-29, 31, 33, 35-36, 38-39, 42-44, 46, 49 and 51-52. The results of post hoc tests demonstrated that the SZ group had significantly lower [deoxy-Hb] concentration changes than the NC group on 16 channels($p < 0.05$, FDR corrected) (Ch. 23, 29, 33-41, 44-46 and 49-50) . The 16 channels comprised the following areas of the brain: right premotor and supplementary motor cortex (SMA) (BA6, Ch. 23), bilateral orbitofrontal cortex (OFC) (BA11, Ch. 35-39, 46， 49-50), bilateral superior temporal gyrus (STG) (BA22, Ch. 33, 41), right temporal gyrus (MTG) (BA21, Ch.44), and bilateral inferior prefrontal gyrus (IFG) (BA47, Ch. 29, 34, 40, 45). We also found that [deoxy-Hb] concentration changes in the task period were markedly lower in the patients with OCD than in the NC group on 5 channels ($p < 0.05$, FDR corrected), including Ch. 28, 35, 38, 46 and 51. The 5 channels comprised the following areas of the brain: bilateral OFC (BA11, Ch. 28, 35, 38 and 46) and left MTG (BA21, Ch.51).

In addition, after FDR correction ($p < 0.05$), the results of post hoc tests showed that there was no significant difference between the OCD and SZ group in [deoxy-Hb] concentration changes in the task period.

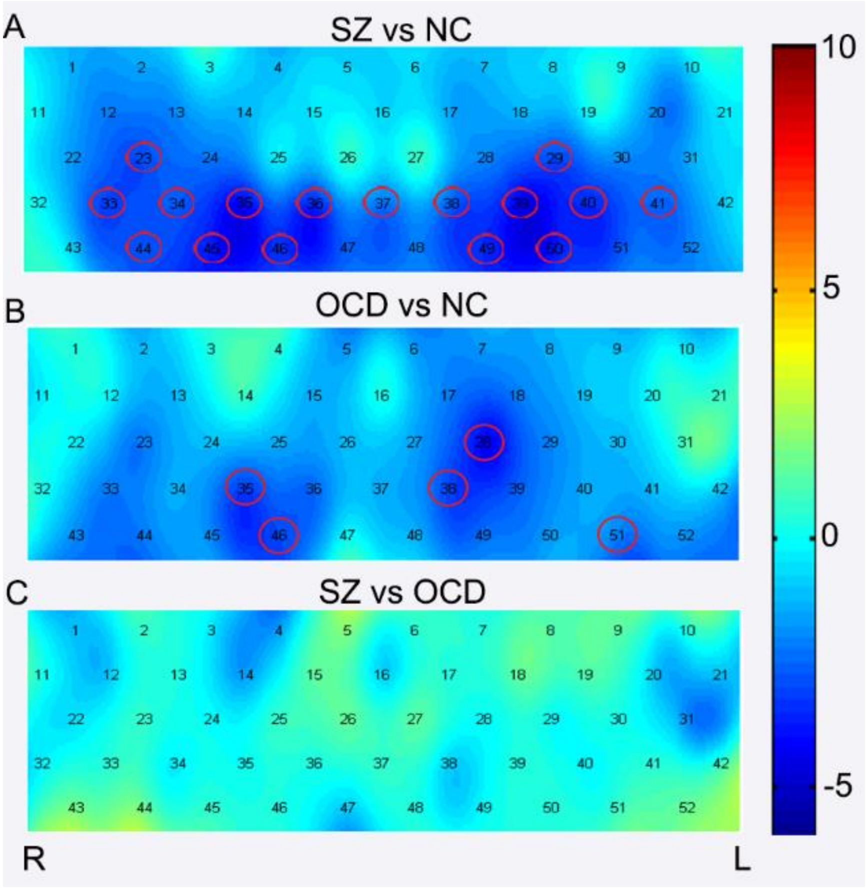

Fig.S1 During VFT, brain activation was measured by alterations in [deoxy-Hb] concentrations in patients with OCD, SZ, and NC. The channels that reached statistical significance were represented by red cycles. $p < 0.05$, FDR correction. Color bar represent t values.

(A): The group comparison for brain activation between the SZ and NC.

(B): The group comparison for brain activation between the OCD patients and NC.

(C): The group comparison for brain activation between the SZ and OCD patients.

SZ, schizophrenia NC, normal controls; OCD, obsessive-compulsive disorder; R, right; L, left.
